# Prevalence of drug use and substance dependence among university students at the University of Girona

**DOI:** 10.1192/j.eurpsy.2024.462

**Published:** 2024-08-27

**Authors:** F. Calvo, C. Giralt, X. Solench-Arco, J. Patiño-Masó, S. Font-Mayolas

**Affiliations:** ^1^Departament de Pedagogia, Institut de Recerca sobre Qualitat de Vida; ^2^Institut Català de la Salut, Centre d’Atenció Primària Blanes 2; ^3^Universitat de Girona; ^4^Departament d’Infermeria; ^5^Departament de Psicologia, Institut de Recerca sobre Qualitat de Vida, Girona, Spain

## Abstract

**Introduction:**

This study examines the prevalence of drug use and substance dependence among university students majoring in Social Education at the University of Girona, aiming to comprehend its impact on the mental health of this population.

**Objectives:**

To determine the prevalence of drug use and substance dependence among university students majoring in Social Education at the University of Girona and to examine gender differences in consumption patterns.

**Methods:**

A cross-sectional, observational, and analytical design was employed. The study population consisted of 258 enrolled students in the program. Convenience sampling was used, with a sample size of 156 students, confidence level of 95%, and margin of error of 5%. The final obtained sample size was n=161. An ad hoc questionnaire was used to collect data on general characteristics and drug use. Statistical analysis included Pearson’s Chi-square tests and Student’s t-tests.

**Results:**

A total of 161 students participated (88.2% females, 11.2% males), with an average age of 21.61 years. Among them, 75.8% grew up in structured families, while 24.2% came from dysfunctional families. Regarding socioeconomic status, 4.3% considered themselves from a low-class background, 32.9% from low-middle class, 51.6% from middle class, and 11.2% from upper-middle class.

Substance dependence was identified in 29.2% of the participants: alcohol (20.3%), MDMA (11.1%), cocaine (10.3%), psychopharmaceuticals (4.8%), and hallucinogenic mushrooms (4.0%). No significant differences were found in SDS scale scores for determining dependence thresholds for any substances except for cannabis (Males = 6.13 vs. Females = 1.80, t = 3.886, df = 83, p < .001). A total of 55.6% of males showed substance dependence compared to 25.7% of females (X^2 = 6.853, df = 1, p = .009).

**Conclusions:**

This study highlights a concerning prevalence of drug use and substance dependence among university students majoring in Social Education at the university, with certain gender-based consumption pattern differences. These findings emphasize the urgency of intervention approaches targeting mental health and substance prevention in this specific population.

**Disclosure of Interest:**

None Declared

